# A simple implantation method for flexible, multisite microelectrodes into rat brains

**DOI:** 10.3389/fneng.2013.00006

**Published:** 2013-07-24

**Authors:** Anja Richter, Yijing Xie, Anett Schumacher, Susanne Löffler, Robert D. Kirch, Jaafar Al-Hasani, Daniel H. Rapoport, Charli Kruse, Andreas Moser, Volker Tronnier, Sandra Danner, Ulrich G. Hofmann

**Affiliations:** ^1^Neurochemistry Group, Department of Neurology, University of LuebeckLuebeck, Germany; ^2^Graduate School for Computing in Medicine and Life Sciences, University of LuebeckLuebeck, Germany; ^3^Neuroelectronic Systems, Department of General Neurosurgery, University Hospital FreiburgFreiburg, Germany; ^4^Department of Psychology, University of Toronto ScarboroughToronto, ON, Canada; ^5^Fraunhofer Institution for Marine Biotechnology, Fraunhofer SocietyLübeck, Germany

**Keywords:** neuroelectrophysiology, brain, implantation, flexible device, rat, deep brain, microprobes

## Abstract

A long term functional and reliable coupling between neural tissue and implanted microelectrodes is the key issue in acquiring neural electrophysiological signals or therapeutically excite neural tissue. The currently often used rigid micro-electrodes are thought to cause a severe foreign body reaction resulting in a thick glial scar and consequently a poor tissue-electrode coupling in the chronic phase. We hypothesize, that this adverse effect might be remedied by probes compliant to the soft brain tissue, i.e., replacing rigid electrodes by flexible ones. Unfortunately, this flexibility comes at the price of a low stiffness, which makes targeted low trauma implantation very challenging. In this study, we demonstrate an adaptable and simple method to implant extremely flexible microprobes even to deep areas of rat's brain. Implantation of flexible probes is achieved by rod supported stereotactic insertion fostered by a hydrogel (2% agarose in PBS) cushion on the exposed skull. We were thus able to implant very flexible micro-probes in 70 rats as deep as the rodent's subthalamic nucleus. This work describes in detail the procedures and steps needed for minimal invasive, but reliable implantation of flexible probes.

## Introduction

Electrical stimulation of discrete brain areas is a clinically increasingly used symptomatic treatment for neurological movement and affective disorders (Schlapfer and Bewernick, 2009; Pizzolato and Mandat, 2012). To further improve this treatment, the necessity arises to optimize the brain-device-interface on the cellular scale (Martens et al., 2010). With respect to this goal, pre-clinically often used microprobes for brain stimulation and recording are tethered to the skull (Hiller et al., 2007) and by nature of their rigidity present strong mechanical provocation to the surrounding tissue (Szarowski et al., 2003; Biran et al., 2007; McConnell et al., 2009). Traditionally implantable electrodes are rigid, thus showing no probe deformation during insertion (Campbell et al., 1991; Rousche and Normann, 1992). Furthermore, clinical implantation equipment provides precise location control to access brain areas specifically (Schjetnan and Luczak, 2011). After implantation, however, electrophysiological connection is impaired e.g., due to chronic gliosis, mechanical trauma or long-term inflammation (Turner et al., 1999; Polikov et al., 2005; Leach et al., 2010).

Recently, flexible microprobes have been developed from different polymers to offset this tissue scar formation by better adapting the probe's mechanical properties to the target tissue (Takeuchi et al., 2005; Kozai and Kipke, 2009; Rubehn and Stieglitz, 2010; Lee et al., 2012; Kim et al., 2013). In contrast to rigid ones, flexible implants are supposed to follow intrinsic movements of the brain resulting from breathing, blood circulation, or body movements (Rousche et al., 2001; Mercanzini et al., 2009; Rubehn and Stieglitz, 2010; Andrei et al., 2012). To test the assumption, we needed to stereotactically implant flexible microprobes in brain structures (Williams, 2008; Hassler et al., 2011). However, thin (~20μm), film-like Polyimide microprobes are impossible to implant like the well-known rigid shank electrodes, since their inherent flexibility results in a buckling force leading to uncontrolled bending still outside the brain tissue. Instead we developed and present here a supported insertion method with the support removed after reaching the target requiring no change in Polyimide probe design. This method enables the insertion of Polyimide-based, ~20μm thick, 350 μm wide and 1.5 cm long electrodes down to the subthalamic nucleus (~8.5 mm) of rat's brain using commercially available stereotactic equipment (David Kopf Instruments, Model 900).

## Materials and methods

See the accompanying video for a visualized version of the protocol. A list of materials required for this procedure can be found in Table 1. We strongly recommend to practice this procedure with already euthanized animals from other experiments prior to *in vivo* application. A competent handling of all instruments is strongly recommended to achieve reproducible results.

**Table 1:** **Specific reagents and equipment required for this approach**.

**Name of the reagent**	**Company**	**Catalog number**
Surgical disposable scalpel #21	Braun Aesculap AG	5518075
Surgical disposable scalpel #23	Braun Aesculap AG	5518016
Cotton swabs, walnut sized	Henry Schein Medical GmbH	9003187
5/0 Premilene-DS16 surgical sutures	Braun Aesculap AG	2090212
Leukofix fixing tape	Henry Schein Medical GmbH	220-544
Tungsten rod ø 140 μm	A-M Systems, Inc.	7166
Loctite 4061 rapid glue	Henkel Loctite GmbH	26085
0.9 mm drill bit	FineScienceTools, USA	19007-09
Rechargeable Cordless Micro Drill	Stoelting Co., USA	58610
Small Animal Stereotaxic Instrument	David Kopf Instruments, USA	Model 900
7.5% betaisodona solution	B. Braun Melsungen AG	3864154
Softasept (74.1% Ethanol and 10% 2-Propanol)	B. Braun Melsungen AG	3887138
Agarose, low gelling temperature (<65°C)	Sigma-Aldrich Co.	A9414
Anaesthetics and other medicine according to approved procedures.		

### Presurgical preparation procedure

Aseptic technique should be used for all survival surgical procedures. Disinfect the surgical work surface with commercial disinfectant and prepare sterile surgical packs of instruments, drapes, gauze, swabs, sutures, and scalpel blades. A surgical mask, hair bonnet and sterile gloves should be worn. A Germinator dry bead sterilizer is also used to re-sterilize surgical instruments between procedures if multiple rat surgeries will be done during one session. Preparation of the rats including disinfection, anesthesia and positioning in the stereotactic frame was performed according to standardized stereotactic procedures, which were reviewed and approved by committees of the University of Lübeck and the responsible Ministry for Agriculture, Environment and Rural Areas, Schleswig-Holstein, Germany.

Adult, male Wistar rats (250–300 g) were anesthetized using an i.p. injection of a Ketamine/Xylazine cocktail [100 mg/kg Ketamine (100 mg/ml), 8 mg/kg Xylazine (2%)]. This amount is generally sufficient for a typical 60 min surgery. If needed, rats can be redosed using a fraction of the original dose. Disinfectant (Softasept) was applied to the scalp starting from the center of the surgical region, spiraling outward and then rinsed with Ringer's solution. The surgical field was prepared by shaving the area using small animal shears. Protective ointment (Bepanthene® nose and eye cream) was applied to the eyes to prevent drying and to provide a physical barrier during surgery. Anesthetized and shaved rats were placed in a stereotactic frame with atraumatic ear bars (tooth bar = 0.0)^1^.

As an insertion tool we clamped a tungsten rod (ø140–175 μm) to the head of a stereotactic arm. The length of the tungsten rod below the clamp head should be ~2 cm to provide sufficient space for manipulations. The rod must be perfectly straight, as can be tested by rolling it on a flat ground.

### Skin incision and preparation of subcutaneous tissue to expose skull

A midline incision of ~1.5 cm length starting between the ears (bregma) and extending toward the nose was made with a scalpel. The subcutaneous tissue was prepared bluntly with two cotton swabs by vigorous but slow rubbing from the center to the periphery of the wound. This stretches the cutaneous and subcutaneous tissues and opens the operation area above the skull. It has to be performed until the necessary operation area is free of all tissues above the skull bone and bleeding has stopped. A well prepared skull is indicated by a typical squeaking sound when rubbing the swab on the bone.

### Widening of the surgical field

A wide, flat operation area is essential for successful implantation. Wound rims were fixed using surgical suture loops (5/0 Premilene-DS16). Loop ends are fixed at the frame with a non-residue tape (Leukofix) while carefully pulling the wound boundaries apart and thus widening the surgical area. That way the desired anatomical orientation points become visible, in our case lambda and the interaural line. We strongly recommend folding the skin on the rostral end by a double stitch which lessens traumatization. If necessary such a loop can be used to fix the caudal end of the wound rim as well.

### Adjustment of the stereotactic frame and targeting

The wound area was washed with Ringer's solution and cleaned with walnut sized cotton swabs prior to targeting. The stereotactic arm is positioned such that the securely mounted insertion tool was moved to the anatomical reference point of choice (lambda in our case). The target coordinates were determined according to the medial:lateral and anterior:posterior axes found in a stereotactic atlas. The insertion arm of the stereotactic frame was positioned over the target point and the tungsten rod was carefully lowered until it touched the skull. The z-position was noted and the target depth was calculated^1^.

### Trepanning the skull

First, the target point was marked on the skull by careful circular nicking the bone with a 20 G needle below the tungsten rod. The produced cavity is used as starting point for drilling—otherwise the skull may be too slippery for precise drilling. When not needed *in situ*, the stereotactic arm was moved out of harms way to carefully maintain insertion rod registration.

A handheld microdrill with a small drill bit (here 0.9 mm ø) was then used to make the burr hole. Drilling was done in short intervals (depending upon the power output of the drill) and drill progress was controlled visually in between. The complete penetration of the skull bone is clearly noticeable by a sudden loss in resistance to the drill bit. Carefully remove it from the drill site and flush all bone fragments with Ringer's solution. Lesser experienced surgeons may want to use a stop above the drill tip by slipping a cylinder, cut from a medical tubing, over the drill. For the price of a slightly diminished view, the drill won't penetrate deeper than the catch allows and thus minimizes uncontrolled brain penetration (Pohl et al., 2011).

### Penetration of the meninges

A successful drilling procedure will leave meninges intact, shimmering opaque at the base of the burr hole. Removing them in a controlled way was done with a slightly nicked 23 G cannula tip. Twisting the cannula‘s now ridgy tip on the base of the hole and pulling it produces a noticeable rupture of the elastic dura mater. Severing the pia mater results in bleeding, which has to be flushed away until it stops. The repetition of this step along the boundary of the hole may be necessary until no more elastic resistance exists. If successfully performed, this step leaves no opaque meninges remaining in the burr hole. This part of the procedure may impair recordings from superficial layers of the cortex.

### Introducing an agarose block into the operation area

All of the steps up until this point are standard for any type of stereotactic intervention in a rat's brain. Next, we introduced a novel tool to facilitate implantation of flexible microprobes. A block of 2 mm height from 2% (w/v) agarose gel was placed over the burr hole on the skull with a sterile spatula. One edge of the block was lodged under the anterior wound fold with close contact to the anterior borders. The posterior part of the gel block extended over the caudal wound boundaries thus increasing the working area. This afforded a slight slope down to the anterior edge of the wound, which facilitated the subsequent microprobe insertion. The airspace between the agar block and the skull was filled by injecting Ringer's solution, which released trapped air bubbles from beneath the surface of the gel block. Ringer's solution was also used to maintain a moist and slippery working surface.

**Figure 1:**
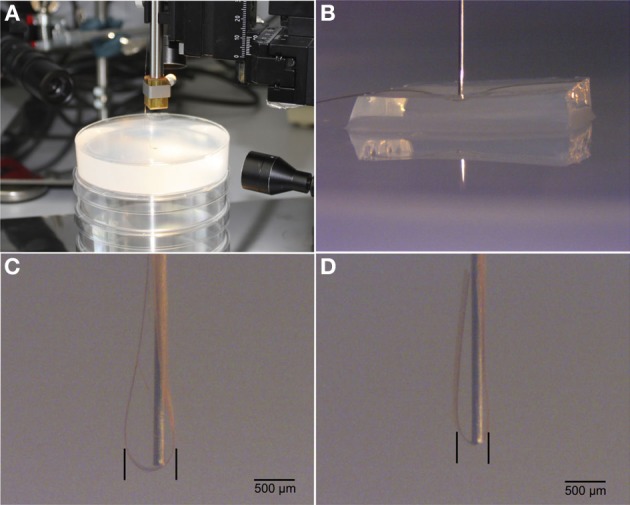
**Illustrations of test insertions of flexible probes into a brain phantom. (A).** Experimental setup including 2% agarose gel. **(B)**. Micrograph of insertion rod on top of flex probe and agarose cushion. **(C)**. Insertion result in brain phantom without agarose cushion. **(D)**. Insertion result with agarose cushion. Note the difference of spread of flex probe around the implantation rod.

Using this agarose gel block improves the positioning of the flexible probes, keeps the exposed brain surface moist, and avoids scratching the probe's surface on skull bone. In addition, the 2% agarose gel block facilitates close wrapping of the probe around the insertion rod. To estimate the effects of using 2% agarose gel in probe insertion, we performed a dummy insertion experiment in a brain-tissue-like phantom (Figures 1A,B). Figures 1C,D. demonstrate the difference of implantation channel width without and with 2% agarose block in a brain phantom (Chen et al., 2004).

### Positioning of the implant and insertion start

**Figure 2:**
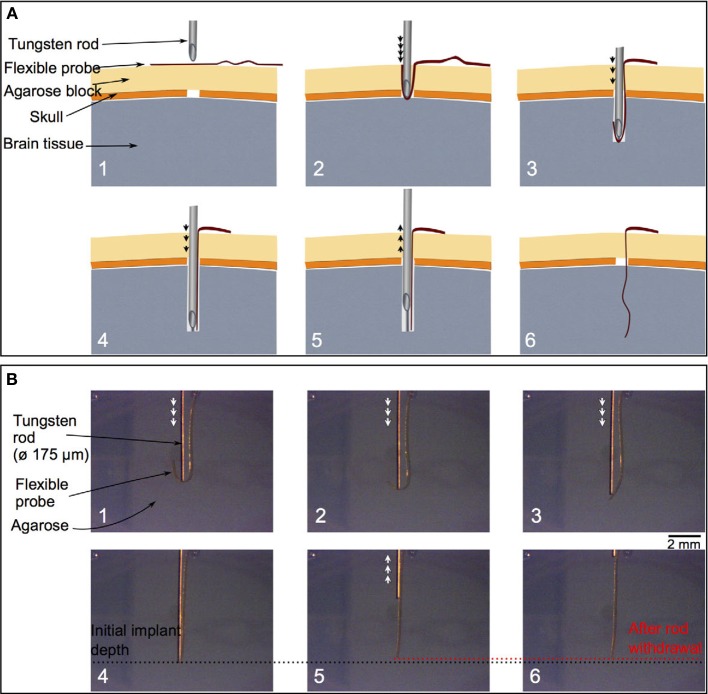
**A schematic cross-section of the insertion field (A).** The arrows indicate the tungsten rod movement direction. Above the brain tissue (gray) the skull and meningeal layer (dark orange) display a burr hole (white space). The tailored agarose cushion (light orange) is used as an insertion matrix for the flexible probe (red) (A: 1). Advancing the insertion tungsten rod (upper grey structure) along the planned trajectory the probe wraps around the rod's tip and is thus arranged by the gel for optimal control (A: 2–4). After the flexible probe is completely straightened, the insertion rod is withdrawn slowly from the remaining probe (A: 5, 6). Image sequence of inserting a flexible probe with a tungsten rod (with flat tip, ø 175 μm) to a 0.5 % agarose gel **(B)**. The arrows indicate the tungsten rod movement direction. During the insertion, the flexible probe first folds and wraps around the rod (B: 1–3). Until it reaches the planned depth position (the black dotted line) and cis ompletely straightened, the insertion rod is withdrawn (B: 4, 6). The red dotted line in panels B5 and B6 indicates the depth position of the flexible probe tip after the rod is removed. It illustrates a misplacement of around 100 μm from the initial depth position.

The flexible microprobe was transferred with a pair of tweezers from its sterile storage onto the gel block, as precise and flat as possible, with the tip positioned 5–6 mm past the insertion site. The stereotactically mounted insertion rod was again positioned above the target point (Figure 2A1). As soon as the tungsten rod is inserted, it pushes the probe into the burr hole (Figures 2A2–A4). The flexible microprobe aligns itself along the rod, thus minimizing cross-section and trauma upon insertion into brain tissue (Figures 2A5,A6). To visualize and evaluate the insertion procedure, we showed here in Figure 2B a sequence of side-view images of probe insertion with a tungsten rod into a translucent brain-tissue-like phantom.

The transparent agarose block allowed for visual control of the relative alignment of the microprobe and the insertion rod. The diffraction at the gel/air boundary may give the appearance of missing the burr hole, which is a known optical illusion. Additional Ringer's solution applied to the gel block minimizes friction, which is essential for maintaining mobility of the microprobe during the insertion procedure. Additional bleedings upon insertion is usually a result of an incomplete disruption of the pia mater and thus must be avoided with proper preparation of the entry hole. Finally, the microprobe was partially inserted by advancing the insertion rod until a desired length of the microprobe remained outside of the skull.

### Removal of the agarose block and insertion finishing

The part of the agarose gel anterior to the insertion rod was cut parallel to the interaural line using a scalpel. The anterior gel block can now be removed by shoving it away from the insertion rod. The microprobe's connector pad, meanwhile, was very gently flipped toward the tungsten rod and thus moved out of harm's way. Subsequently, the posterior triangle of the gel is shoved out of the wound area. Manipulation of agarose gel was accomplished with a sharp scalpel tip, as it is too slippery for forceps, but easily pinned down by the blade. After the agarose block had been removed completely, the microprobe insertion was finalized until the desired target depth was reached.

### Mounting of the implant

Remaining Ringer's solution was removed and the skull thoroughly dried using cotton swabs. The microprobe was fixed to the skull using a small spot of medical super glue (Loctite) deposited at least 5 mm posterior to the burr hole. The connector pad was pushed down into the glue using a scalpel tip. Forceps are not recommended here, since the glue may rapidly fix their arms shut, which will destroy the entire implantation upon the ensuing struggle to free them. A scalpel, however, easily cuts itself free through gentle twisting.

The connector pad was covered with another drop of glue and upon solidifying, the tungsten rod was removed from the brain by smooth and continuous upward movements. The entire area is flushed clean with Ringer's solution, which polymerizes all leftover super glue. A pair of forceps were used to carefully scrape all inadvertently fixed connective tissue free.

The wound closure may be performed according to the experimental needs. In our histological study we sutured the skin over the implantation site, cleaned the suture with Ringer's solution and applied iodide disinfectant and local anesthetic. The animal was monitored and kept warm on a temperature controlled plate until it was fully awake.

## Results

The most significant result of this method is the successful implantation of very flexible, film-like microprobes into deep brain regions with minimal tissue disruption. Cross-sectional histology demonstrated that we reliably reached our target point deep in the brain of rats.

**Figure 3:**
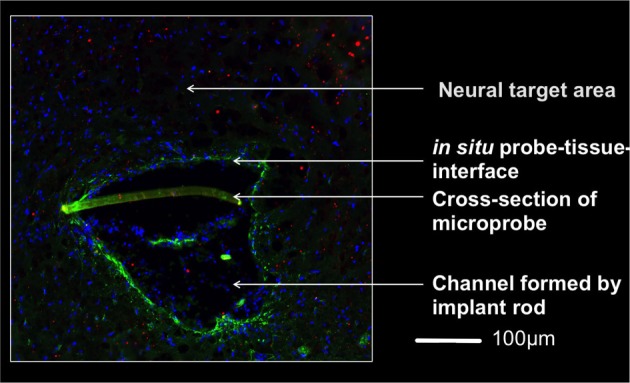
**Micrograph of the implantation area one week after implantation 4,5 mm below the brain surface.** The rod supported insertion of the microprobe yields an implantation channel on one side of the slightly crescent-shaped probe (350 μm wide). Invading cells to this region are indicated by stained nuclei (DAPI, blue). A thin glial layer (glial fibrillary acidic protein, green) surrounds the lesion and reflects a mild tissue reaction. The lack of a pronounced extracellular matrix layer (chondroitinsulfate proteoglycan, red) supports this observation. The brain-probe-interface showed no cysts. Magnification 20x with an PlanApochromat-Objective at AxioObserver Z.1 (Carl Zeiss MicroImaging GmbH, Germany).

**Figure 4:**
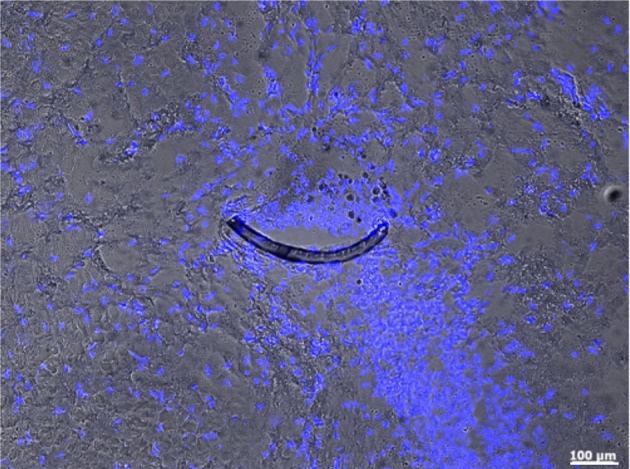
**Microprobe cross-section (crescent-shaped strip) in an horizontal slice of the subthalamic nucleus of an implanted rat 4 weeks after surgery.** This micrograph combines fluorescence and bright-field illumination, where nuclei (blue) are stained with DAPI. Magnification 10x with an PlanApochromat-Objective using an AxioObserver Z.1 (Carl Zeiss MicroImaging GmbH, Germany).

One week after surgery we observed in the implanted rats the inevitable lesion caused by the insertion needle next to the implant (Figure 3). The tissue reaction was evident from a thin glial layer homogeneously surrounding the implantation lesion. A clear, but thin brain-probe-interface without cysts is indicative for a good connectivity to the target area adjacent to the implant. After four weeks a cyst-free tissue next to the implantation-site was visible (the central free space is the original position of the implant, moved upon slicing), indicating a good mechanical connectivity of the implant to the target area (Figure 4).

**Figure 5:**
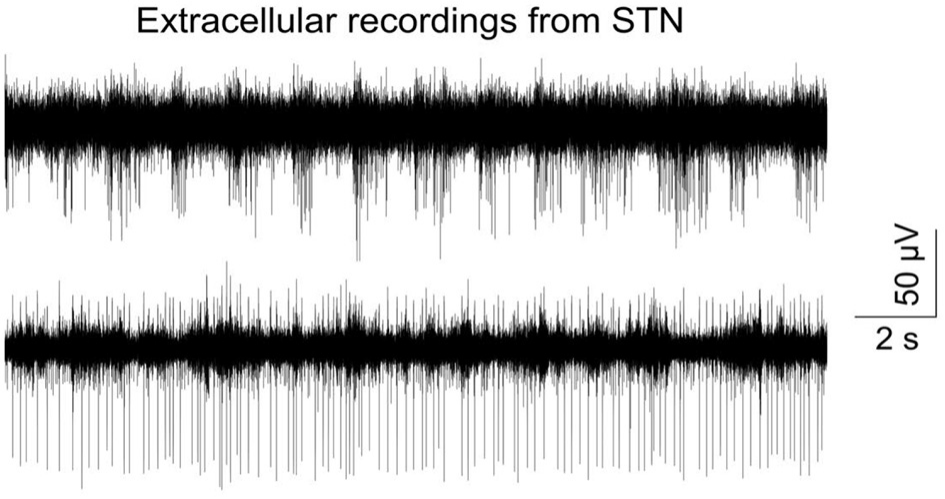
**Typical signal trace recorded from the subthalamic nucleus right after insertion of a flexible microprobe; bandpass filtered (400–4000 kHz) and sampled with 24414 kHz.** Recording was done with an electrical connected flexible microprobe as described in Löffler et al. (2012).

High precision and successful implantation was corroborated by recording electrophysiological signals from neuronal tissue adjacent to the target area (subthalamic nucleus) immediately following implantation (Figure 5) and four weeks post-surgery with flex probes of slightly different design. Detailed histological data will be published elsewhere. Seventy animals have undergone this procedure to date and all of them survived without incident until the planned euthanasia six months following surgery.

## Discussion

Although the hypothesis on the beneficial effect of the probe's elasticity matching the brain's was mentioned more than a decade ago (Stieglitz and Meyer, 1999), all efforts to use flexible probes on a large scale were less efficient due to the difficulty to implant them in a reliable way in deeper brain regions (Rousche et al., 2001; Kozai and Kipke, 2009). We thus started with an established procedure for implantation of rigid electrodes and modified it to our needs. The animals implanted with our technique all survived the implantation without exception. No abnormalities of movements and/or behavior were developed post operatively. Histological examination confirmed the positioning of the implant in the target area with minimal tissue disruption. To ensure reproducibility and best possible use of limited time under anesthesia, we strongly recommend to exercise this procedure with agarose dummies or dead animals before starting on living ones. There are several finely tuned handling steps, which require sensitive use of instruments, microprobe and surgical technique. A well experienced experimenter will need less than one hour for the entire procedure, for which the initial dose of Ketamine/Xylazine-cocktail may not quite suffice. Deep anesthesia has to be maintained at all times by re-injecting fractions of the initial dose.

The extremely cautious use of the miniature power drill and the subsequent removal of the meninges is essential for exerting minimal tissue damage. An automatized drill system may reduce the risk of severe brain trauma (Pohl et al., 2012).

However, in our view the highest optimization potential remaining is the design of the insertion tool/microprobe connection. This study was based on temporarily fixating both by dynamic forces, a removable anchoring of the probe to the insertion tool might improve positioning even further as was suggested by Löffler et al. (2012). Another field of needed improvement is the electrical connection of the probe to the periphery in response to wound closure procedures and probe specifications. However, for our histological study tethering by super glue (Loctite) proved sufficient. We were thus able to demonstrate with a simple method the repeatable implantation of these flexible electrodes to deep brain areas of rats.

## Conflict of interest statement

The authors declare that the research was conducted in the absence of any commercial or financial relationships that could be construed as a potential conflict of interest.

## Footnotes

^1^One of the reviewers recommends for improved animal welfare the following steps in addition to the demonstrated ones:

- Before securing ears in earbars, apply Antipyrine-Benzocaine otic solution to ears.
- For monitoring depth of anesthesia and overall animal health, it is helpful to insert rectal thermometer probe.
- Removal of subcutaneous tissue can be done more effectively by cutting the muscle with the scalpel and scraping periosteum with a Freer chisel while controlling the skull bleeding with bone wax.
- After drilling the skull and before puncturing the meninges, it is suggested to apply Sensorcaine (bupivacaine with epinephrine) to the cortex to produce local anesthesia and vasospasms (to reduce cortical bleeding).
